# Inflammatory parameters mediates the relationship between dietary index for gut microbiota and frailty in middle-aged and older adults in the United States: findings from a large-scale population-based study

**DOI:** 10.3389/fnut.2025.1553467

**Published:** 2025-04-16

**Authors:** Qijiang Yang, Xiaoyun Wu, Jinlan Duan, Yiyin Chen, Tianrui Yang

**Affiliations:** ^1^Department of General Medicine, The First People's Hospital of Zhaoqing, Zhaoqing, Guangdong, China; ^2^Department of Geriatrics, The First People's Hospital of Yunnan Province, Affiliated Hospital of Kunming University of Science and Technology, Kunming, China; ^3^Division of Geriatric Medicine and Gerontology, Johns Hopkins University School of Medicine, Baltimore, MD, United States

**Keywords:** dietary index for gut microbiota, DI-GM, frailty, inflammation, NHANES

## Abstract

**Background:**

Frailty is a prevalent geriatric syndrome marked by diminished physiological reserves and heightened vulnerability to stressors, leading to adverse health outcomes and imposing significant economic burdens on healthcare systems.

**Methods:**

This study investigates the relationship between the Dietary Index for Gut Microbiota (DI-GM) and the risk of frailty in middle-aged and older adults, using data from the National Health and Nutrition Examination Survey (NHANES) collected from 2007 to 2018. Weighted logistic regression, subgroup analysis, and restricted cubic splines (RCS) were performed to evaluate the relationship between DI-GM and frailty risk. Additionally, a mediation analysis was conducted to investigate the influence of relevant inflammatory parameters from complete blood count, including leukocyte count, neutrophil count, the neutrophil to lymphocyte ratio (NLR), and the systemic inflammatory response index (SIRI), to elucidate how DI-GM may influence the onset and progression of frailty.

**Results:**

In this cross-sectional analysis of 8,695 participants with a mean age of 65.56 years, 3,173 individuals were classified as frail. After adjusting for all covariates, a significant inverse relationship was observed between DI-GM and the risk of frailty. Quartile analysis revealed that participants in the highest quartile of DI-GM had significantly lower odds of frailty compared to those in the lowest quartile (OR: 0.80, 95% CI: 0.65–0.99, *p* = 0.04). Trend analyses across all models demonstrated a consistent inverse relationship between higher DI-GM quartiles and frailty odds (*p* < 0.0001 for the crude model; *p* = 0.001 for Model 1; *p* = 0.04 for Model 2). Subgroup analyses confirmed the stability of the impact of DI-GM on frailty risk across various subgroups. RCS showed that the risk of frailty decreased linearly with increasing DI-GM levels. Mediation analysis indicated significant effects for leukocyte count, neutrophil count, NLR, and SIRI, with mediation proportions of 5.7, 7.9, 4.4, and 5.5%, respectively (all *p* < 0.001).

**Conclusion:**

The levels of DI-GM are inversely associated with the risk of frailty, with part of this association mediated by inflammatory parameters.

## Introduction

Frailty is a multifactorial clinical syndrome predominantly observed in older adults, characterized by a decline in physiological reserves and increased vulnerability to stressors. This syndrome is associated with significant morbidity, diminished quality of life, and elevated mortality rates in the elderly population (1). A growing body of literature emphasizes the importance of identifying modifiable risk factors to prevent the onset and progression of frailty. Current therapeutic modalities, including physical rehabilitation and pharmacological interventions, often exhibit limited efficacy and accessibility (2, 3), highlighting the need for novel strategies in frailty management. In this context, dietary habits emerge as a critical area of investigation, as nutrition plays a fundamental role in maintaining health and resilience in older adults (4). Consequently, there is an urgent demand for research exploring the interplay between dietary habits and frailty risk.

Recent studies have begun to investigate the complex relationship between dietary habits and frailty, particularly focusing on the role of gut microbiota in mediating health outcomes. The gut microbiome, a complex community of microorganisms residing in the gastrointestinal tract, is significantly influenced by dietary patterns (5, 6). Emerging evidence suggests that dietary quality may correlate with the composition and diversity of gut microbiota, which, in turn, may affect various health outcomes, including frailty (7, 8). The dietary index for gut microbiota (DI-GM) has been proposed as a potential tool for assessing dietary quality in relation to gut health (9, 10). Recently, the relationship between dietary quality and human health problems has been studied using DI-GM. For example, studies have shown that DI-GM is significantly associated with biological age, with higher DI-GM scores associated with a lower risk of accelerated aging (11). DI-GM has also been used to study the relationship between diet and health outcomes such as stroke, depression, constipation, and metabolic dysfunction-associated fatty liver disease, further validating its potential to assess the health effects of diet (10, 12–14). While DI-GM has demonstrated significant potential in identifying the relationships between dietary quality and various health outcomes, its specific contributions to frailty remain largely underexplored. Therefore, investigating the relationship between DI-GM and frailty represents an important avenue for future research.

Despite the increasing literature linking nutrition and frailty, significant gaps persist in our understanding of how dietary interventions can effectively reduce the risk of frailty. Previous studies have predominantly focused on specific dietary components or patterns, often neglecting the broader context of gut microbiota interactions (15, 16). Furthermore, potential mediating factors, such as inflammatory responses and metabolic changes associated with dietary intake, have not been adequately addressed. This underscores the necessity for comprehensive research that integrates dietary assessment with a focus on gut microbiome dynamics and inflammatory biomarkers.

To investigate these relationships further, this study employs a cross-sectional analysis utilizing data from the National Health and Nutrition Examination Survey (NHANES) spanning from 2007 to 2018. This dataset provides a robust and nationally representative sample, facilitating a nuanced exploration of the associations between DI-GM and frailty risk in middle-aged and older adults. By employing rigorous statistical methods, this research aims to assess the relationship between dietary quality, as indicated by DI-GM, and frailty risk, while also examining potential mediating factors such as relevant inflammatory parameters from complete blood count.

The specific objectives of this research are as follows: first, to evaluate the relationship between DI-GM and frailty risk in middle-aged and older adults, and second, to investigate potential mediating factors, including inflammatory biomarkers, that may elucidate the pathways through which dietary quality impacts frailty. Previous studies have indicated that inflammation plays a critical role in the frailty phenotype, suggesting that a deeper understanding of these inflammatory pathways could enhance targeted interventions aimed at reducing frailty through dietary modifications.

In summary, this study seeks to contribute to the existing literature by elucidating the relationship between DI-GM and frailty risk among middle-aged and older adults. By integrating dietary assessment with health outcomes and inflammatory markers, we aim to provide insights that may inform future interventions and policies aimed at mitigating frailty through enhancements in dietary quality (17). The findings from this research hold the potential to improve individual health outcomes and alleviate the broader societal and economic impacts of frailty in aging populations.

## Methods

### Data source and participants

The data for this study were obtained from NHANES conducted by the Centers for Disease Control and Prevention, aimed at assessing the health and nutritional status of non-institutionalized civilians in the United States (18). NHANES employs a complex, stratified, multi-stage probability sampling design, with data collected through in-home interviews, dietary assessments, medical examinations, and laboratory tests. All protocols were approved by the National Center for Health Statistics Ethics Review Board, and written informed consents were obtained from participants.

**Figure 1:**
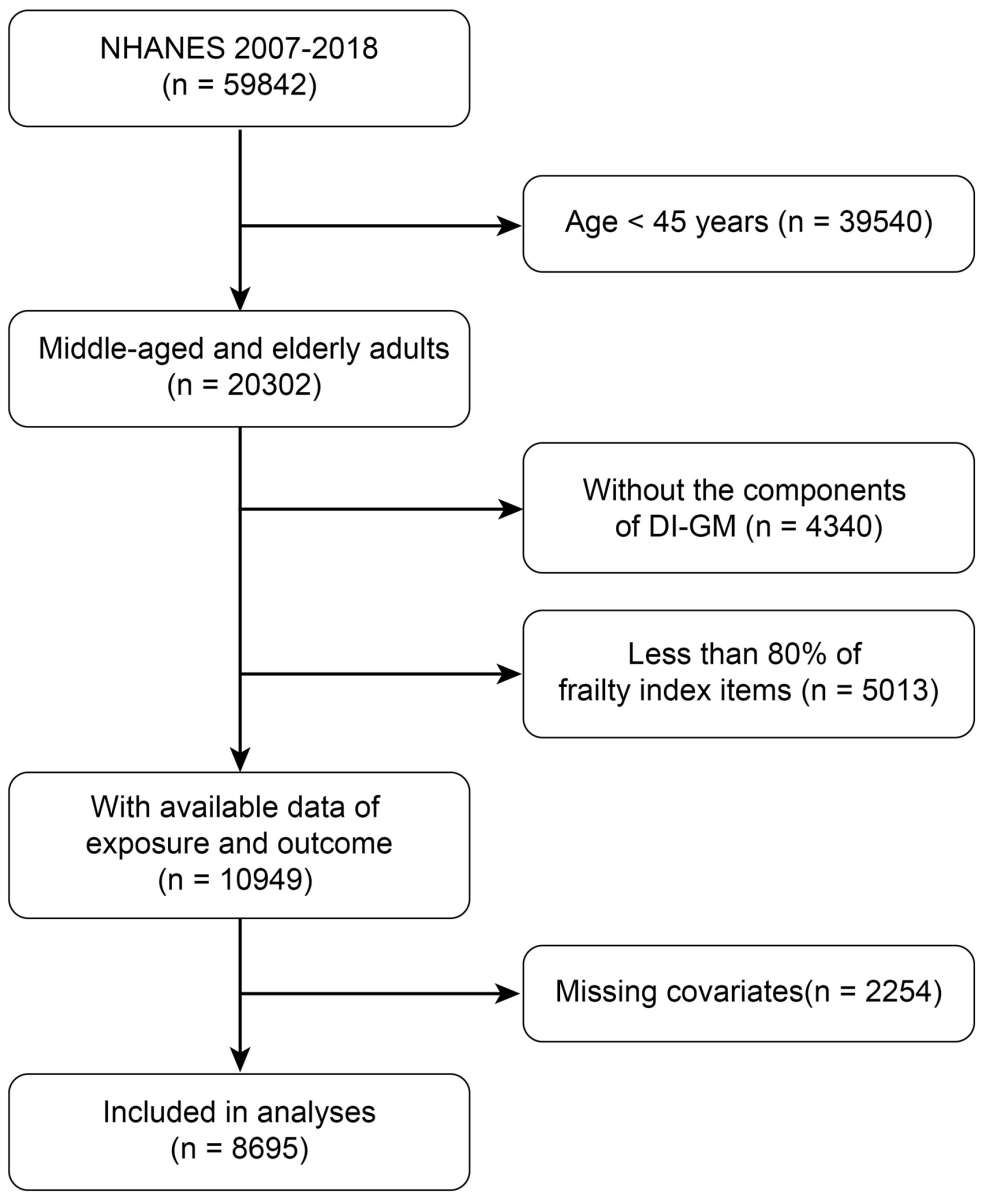
Flow charts for selecting eligible participants from NHANES.

This study utilized data from NHANES covering the period from 2007 to 2018 to investigate the relationship between DI-GM and the risk of frailty in middle-aged and older adults. The initial analysis included a total of 59,842 participants. The following participants were excluded: those aged less than 45 years (*n* = 39,540), those with missing DI-GM component data (*n* = 4,340), those with less than 80% of frailty index items (*n* = 5,013), and those with missing covariate data (*n* = 2,254). The final analysis included 8,695 eligible participants (Figure 1).

### Assessment of dietary index for gut microbiota

This study employed the scoring system developed by Kase et al. to calculate DI-GM, which is based on 14 food items or nutrients (9). Ten beneficial components included avocado, broccoli, chickpeas, coffee, cranberries, fermented dairy, fiber, green tea, soybean, and whole grains, while 4 Unfavorable components comprised red meat, processed meat, refined grains, and a high-fat diet (≥40% of energy from fat). Each component was scored according to gender-specific median intake: for beneficial components, a score of 1 was assigned if consumption met or exceeded the median, otherwise 0; for adverse components, a score of 0 was assigned if consumption met or exceeded the median or the high-fat threshold, otherwise 1. The overall DI-GM score was calculated by summing the scores of all components. In our study, DI-GM was derived by averaging the intakes reported in two 24-h dietary recall interviews. Detailed information on the components and scoring criteria of the DI-GM is provided in Supplementary Table 1.

### Assessment of frailty

Frailty was the outcome variable of this study. This study utilized a modified frailty index developed by Hakeem et al., which is based on 49 items (19). These items assessed deficits across multiple aspects, including cognition, dependence, depressive symptoms, comorbidities, healthcare utilization and access to care, physical performance, anthropometry, and laboratory values. The number of deficits identified for each participant was divided by the total number of considered deficits to calculate the frailty index score, which ranges from 0 to 1. In line with previous studies, we set strict inclusion criteria for frailty diagnosis, requiring participants to complete at least 80% of the index items (at least 40 out of 49) to ensure reliability (20–24). In the descriptive analysis, a cut-off point of 0.21 was used to indicate frailty (19, 25, 26). Detailed information on the variables included in the 49-item frailty index and their respective scoring criteria is provided in Supplementary Table 2.

### Covariates

In our study, covariates were meticulously selected based on prior research and clinical relevance. The demographic variables included age, gender, race, educational level, and the family poverty income ratio (PIR). In the logistic regression analysis, age was treated as a continuous variable. However, for the purposes of describing participant characteristics and conducting subgroup analyses, it was categorized into two groups: 45–64 years and ≥ 65 years. Race was classified into the following categories: Mexican American, non-Hispanic White, non-Hispanic Black, non-Hispanic Asian, and other races. Educational attainment was grouped into three levels: less than high school, high school, and more than high school. The PIR served as an indicator of family income, stratified into three categories: ≤1.3, 1.3–3.5, and >3.5 (22). Lifestyle factors included smoking status, categorized as never, former, and current smokers, and drinking status, classified as never, former, mild, moderate, and heavy drinkers. Recreational activity was assessed and categorized into moderate, vigorous, and other levels of physical activity. Comorbidities were recorded as binary variables (yes/no) for conditions such as cardiovascular disease (CVD), hyperlipidemia, chronic obstructive pulmonary disease (COPD), and chronic kidney disease (CKD). Relevant inflammatory parameters from complete blood count included leukocyte count, neutrophil count, monocyte count, lymphocyte count, platelet count, the neutrophil to lymphocyte ratio (NLR), monocyte to lymphocyte ratio (MLR), platelet to lymphocyte ratio (PLR), systemic immune inflammation index (SII), and systemic inflammation response index (SIRI). The formula for calculating SII is platelet count × neutrophil count/lymphocyte count (27). The formula for calculating SIRI is monocyte count × neutrophil count/lymphocyte count (28).

### Statistical analysis

In line with the multistage sampling framework of NHANES, our analysis incorporated sample weights and strata to account for the complexity inherent in the survey design. Continuous variables were presented as weighted means with standard errors (SE), while categorical variables were presented as weighted percentage. Comparative analyses for these variables were performed using t-tests and chi-squared tests, respectively. We used logistic regression analysis to assess the association between DI-GM and risk of frailty in three models: the crude model without adjustment, model 1 adjusted for age, gender, race, educational level and PIR, and model 2 further adjusted for smoking status, drinking status, recreational activity, CVD, hyperlipidemia, COPD and CKD. To elucidate the relationship between DI-GM and frailty, subgroup analyses were performed to stratify by key demographic and lifestyle factors and comorbidities, including age, gender, race, educational level, PIR, smoking status, drinking status, recreational activity, CVD, hyperlipidemia, COPD and CKD. We also used restricted cubic splines (RCS) to assess the potential non-linear relationship between DI-GM and risk of frailty. Mediation analysis was performed to explore the potential mediating role of relevant inflammatory parameters from complete blood count, including leukocyte, neutrophil, NLR and SIRI, in the association between DI-GM and frailty. All statistical analyses were performed in R (version 4.4.2). A 2-tailed *p* < 0.05 was considered statistically significant.

## Results

### Characteristics of the participants

Table 1 shows the characteristics of a population of 8,695 adults aged 45 years and older in the United States, with a mean age of 65.56 years (SE = 0.18). Of these, 3,173 were classified as frail. Notably, people with frailty were more likely to be older (over 65 years), female, and to have lower levels of education and income. They also had a higher prevalence of comorbid conditions, including CVD, CKD and COPD. Frail people also had elevated inflammatory markers, with significantly higher leukocyte and neutrophil counts, and their GI-DM scores were lower than those of their non-frail counterparts.

**Table 1:** Comparison of baseline characteristics between participants with frailty and those without frailty.

Variable	Total (*n* = 8,695)	Non-frailty (*n* = 5,522)	Frailty (*n* = 3,173)	*P*-value
n (%) ^a^				
Age				0.09
45–64	3,666 (46.58)	2,266 (45.61)	1,400 (48.59)	
≥ 65	5,029 (53.42)	3,256 (54.39)	1773 (51.41)	
Gender				< 0.001
Female	4,446 (54.30)	2,682 (52.26)	1764 (58.54)	
Male	4,249 (45.70)	2,840 (47.74)	1,409 (41.46)	
Race				< 0.0001
Mexican American	963 (4.45)	603 (3.82)	360 (5.74)	
Non-Hispanic White	4,559 (77.79)	2,928 (80.65)	1,631 (71.86)	
Non-Hispanic Black	1,749 (8.76)	1,050 (7.35)	699 (11.70)	
Other Hispanic	847 (3.63)	541 (3.16)	306 (4.61)	
Other Race	577 (5.37)	400 (5.02)	177 (6.09)	
Educational level				< 0.0001
< High school	2,277 (16.31)	1,198 (12.15)	1,079 (24.95)	
High school	2,127 (24.99)	1,302 (23.16)	825 (28.79)	
> High school	4,291 (58.69)	3,022 (64.69)	1,269 (46.25)	
PIR				< 0.0001
≤1.3	2,721 (20.79)	1,325 (14.07)	1,396 (34.72)	
1.3–3.5	3,466 (37.87)	2,203 (36.56)	1,263 (40.60)	
> 3.5	2,508 (41.34)	1,994 (49.37)	514 (24.68)	
Smoking status				< 0.0001
Never	4,066 (48.34)	2,778 (52.41)	1,288 (39.88)	
Former	3,210 (36.29)	2016 (36.21)	1,194 (36.46)	
Now	1,419 (15.37)	728 (11.37)	691 (23.66)	
Drinking status				< 0.0001
Never	1,354 (12.55)	858 (12.93)	496 (11.76)	
Former	2,132 (20.73)	1,096 (16.39)	1,036 (29.71)	
Mild	3,374 (43.90)	2,376 (47.60)	998 (36.23)	
Moderate	987 (13.00)	682 (14.08)	305 (10.76)	
Heavy	848 (9.83)	510 (9.00)	338 (11.54)	
Recreational activity				< 0.0001
Moderate	2,576 (32.77)	1,973 (37.84)	603 (22.25)	
Vigorous	233 (2.55)	190 (3.18)	43 (1.25)	
Other	5,886 (64.68)	3,359 (58.99)	2,527 (76.50)	
CVD				< 0.0001
Yes	2033 (21.57)	654 (11.50)	1,379 (42.45)	
No	6,662 (78.43)	4,868 (88.50)	1794 (57.55)	
Hyperlipidemia				0.01
Yes	7,274 (84.93)	4,522 (83.78)	2,752 (87.33)	
No	1,421 (15.07)	1,000 (16.22)	421 (12.67)	
COPD				< 0.0001
Yes	848 (9.35)	373 (6.74)	475 (14.77)	
No	7,847 (90.65)	5,149 (93.26)	2,698 (85.23)	
CKD				< 0.0001
Yes	2,833 (28.73)	1,389 (22.30)	1,444 (42.05)	
No	5,862 (71.27)	4,133 (77.70)	1729 (57.95)	
DI-GM				< 0.0001
Q1 [0,4]	3,164 (32.95)	1842 (30.46)	1,322 (38.12)	
Q2 (4,5]	1925 (22.79)	1,213 (22.47)	712 (23.45)	
Q3 (5,6]	1,641 (19.23)	1,079 (19.40)	562 (18.89)	
Q4 (6,11]	1,965 (25.03)	1,388 (27.67)	577 (19.54)	
Mean ± SE ^b^				
Age, year	65.56 ± 0.18	65.84 ± 0.20	64.99 ± 0.26	0.004
Leukocyte, 10^9^/L	7.25 ± 0.05	6.97 ± 0.05	7.84 ± 0.12	< 0.0001
Neutrophil, 10^9^/L	4.34 ± 0.03	4.17 ± 0.04	4.69 ± 0.05	< 0.0001
Monocyte, 10^9^/L	0.59 ± 0.00	0.58 ± 0.01	0.62 ± 0.01	< 0.0001
Lymphocyte, 10^9^/L	2.07 ± 0.03	1.97 ± 0.02	2.27 ± 0.10	0.003
Platelet, 10^9^/L	234.20 ± 1.41	232.11 ± 1.46	238.53 ± 2.61	0.02
NLR	2.41 ± 0.02	2.32 ± 0.02	2.59 ± 0.04	< 0.0001
MLR	0.32 ± 0.00	0.32 ± 0.00	0.33 ± 0.00	0.001
PLR	128.96 ± 1.20	129.23 ± 1.27	128.40 ± 2.08	0.7
SII	563.62 ± 7.22	539.56 ± 7.77	613.54 ± 12.25	< 0.0001
SIRI	1.43 ± 0.02	1.34 ± 0.02	1.60 ± 0.03	< 0.0001
DI-GM	5.29 ± 0.04	5.40 ± 0.05	5.05 ± 0.05	< 0.0001

CKD, chronic kidney disease; COPD, chronic obstructive pulmonary disease; CVD, cardiovascular disease; DI-GM, dietary index for gut microbiota; MLR, monocyte to lymphocyte ratio; NLR, neutrophil to lymphocyte ratio; PLR, platelet to lymphocyte ratio; PIR, family poverty income ratio; SE, standard errors; SII, systemic immune inflammation index; SIRI, systemic inflammation response index.

a Sample size (weighted percentage).

b Weighted means ± standard errors (SE).

### Association between dietary index for gut microbiota and frailty

**Figure 2:**
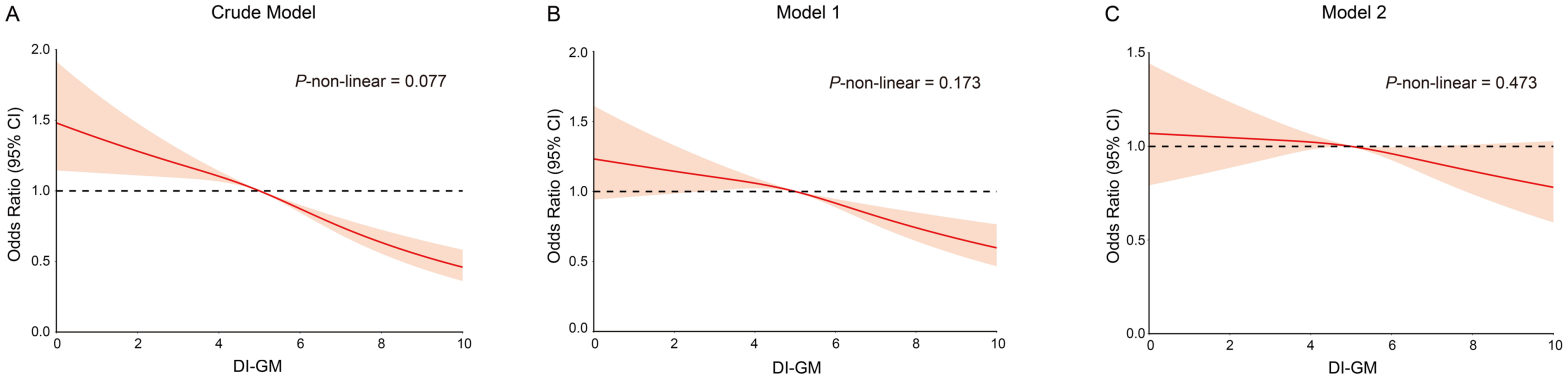
The restricted cubic spline curves for the association between DI-GM and frailty. **(A)** Restricted cubic spline for crude model. **(B)** Restricted cubic spline for model 1. **(C)** Restricted cubic spline for model 2. DI-GM, dietary index for gut microbiota. Crude model adjust for: none; Model 1 adjusted for: age, gender, race, educational level and PIR; Model 2 adjusted for: age, gender, race, educational level, PIR, smoking status, drinking status, recreational activity, CVD, Hyperlipidemia, COPD and CKD. DI-GM, Dietary index for gut microbiota.

Table 2 demonstrates a significant association between DI-GM and the odds of frailty. In the crude model, each unit increase in the DI-GM was associated with a reduction in frailty odds (OR: 0.89, 95% CI: 0.86–0.94, *p* < 0.0001). Model 1, adjusted for demographic factors, continued to show reduced odds (OR: 0.94, 95% CI: 0.90–0.99, *p* = 0.01), while model 2, fully adjusted, showed a nonsignificant association (OR: 0.97, 95% CI: 0.93–1.01, *p* = 0.17). Although the *p*-value exceeds the conventional threshold of 0.05, the point estimate of 0.97 suggests a slight decrease in odds. Quartile analysis revealed that participants in the highest quartile (Q4) exhibited a significantly lower risk of frailty compared to those in the lowest quartile (Q1), (OR: 0.56, 95% CI: 0.47–0.68, *p* < 0.0001). Adjustments in model 1 resulted in a slight attenuation of this association for Q4 (OR: 0.72, 95% CI: 0.59–0.88, *p* = 0.001). Despite this attenuation, model 2 maintained a significant finding for Q4 (OR: 0.80, 95% CI: 0.65–0.99, *p* = 0.04). Trend analyses across all models revealed a consistent inverse relationship between higher DI-GM quartiles and frailty odds (*p* < 0.0001 for the crude model; *p* = 0.001 for model 1; *p* = 0.04 for model 2), supporting the hypothesis that a higher DI-GM may confer protection against frailty. The restricted cubic spline regression model was used to examine the relationship between DI-GM and frailty, revealing an inverse association: higher DI-GM scores were correlated with lower frailty. Analysis across all models indicated no significant nonlinearity, with *p*-values for nonlinearity of 0.077 for the Crude model, 0.173 for Model 1, and 0.473 for Model 2 (Figures 2A–C).

**Table 2:** Association between dietary index for gut microbiota and frailty.

	Crude model		Model 1		Model 2	
	OR (95%CI)	*P*	OR (95%CI)	*P*	OR (95%CI)	*P*
DI-GM	0.89 (0.86, 0.94)	< 0.0001	0.94 (0.90, 0.99)	0.01	0.97 (0.93, 1.01)	0.17
DI-GM						
Q1	Ref		Ref		Ref	
Q2	0.83 (0.69, 1.01)	0.06	0.95 (0.78, 1.17)	0.66	1.00 (0.80, 1.24)	0.99
Q3	0.78 (0.64, 0.95)	0.01	0.91 (0.74, 1.12)	0.37	0.96 (0.76, 1.22)	0.76
Q4	0.56 (0.47, 0.68)	< 0.0001	0.72 (0.59, 0.88)	0.001	0.80 (0.65, 0.99)	0.04
P for trend		< 0.0001		0.001		0.04

DI-GM, dietary index for gut microbiota; OR, odds ratio; CI, confidence interval; Crude model adjust for: none; Model 1 adjusted for: age, gender, race, educational level and PIR; Model 2 adjusted for: age, gender, race, educational level, PIR, smoking status, drinking status, recreational activity, CVD, Hyperlipidemia, COPD and CKD.

### Subgroup analysis

**Figure 3:**
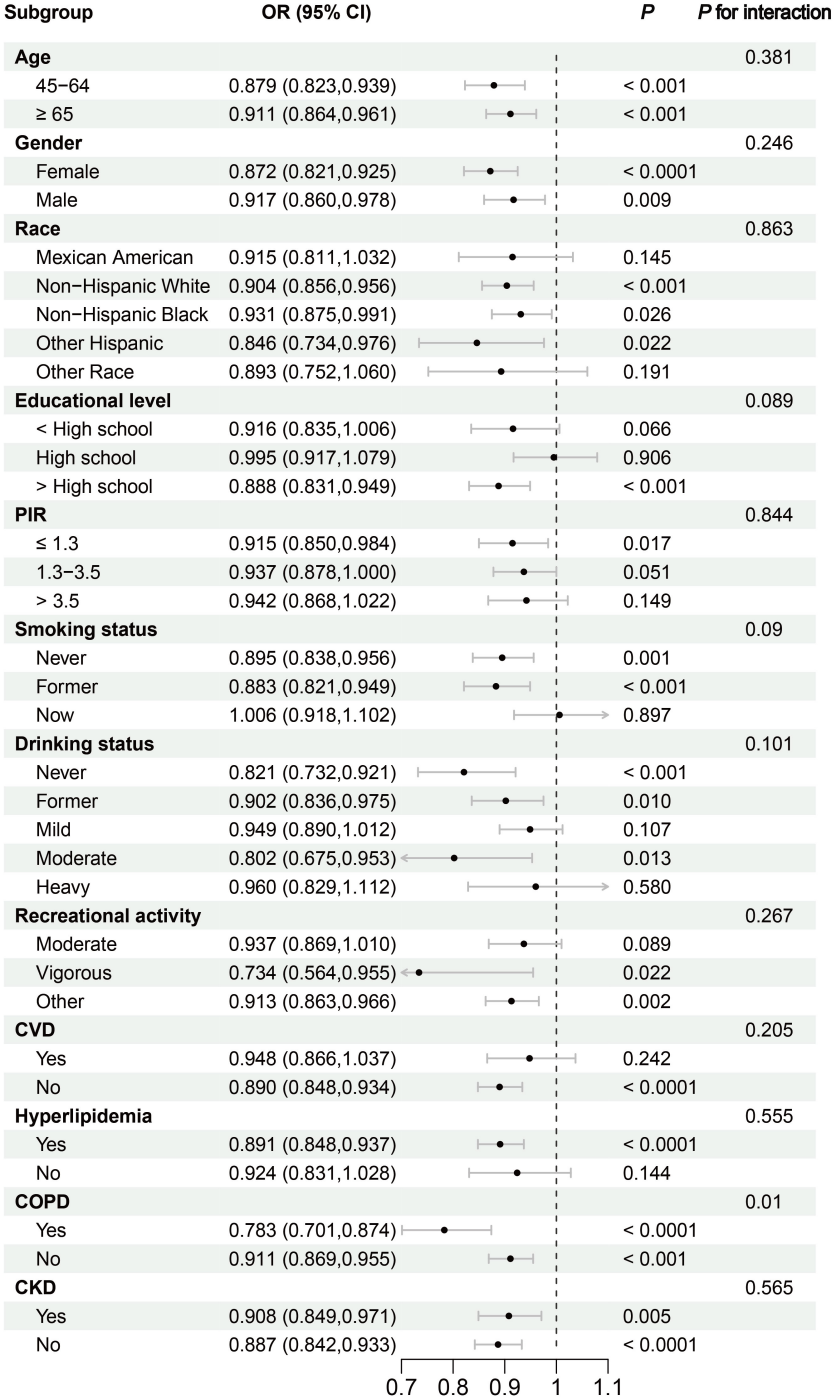
Subgroup analysis of the association between Dietary Index for Gut Microbiota and Frailty. CKD, chronic kidney disease; COPD, chronic obstructive pulmonary disease; CVD, cardiovascular disease; PIR, family poverty income ratio.

Subgroup analyses revealed significant reductions in frailty risk associated with DI-GM across various age groups: individuals aged 45–64 years (OR: 0.879, 95% CI: 0.823–0.939, *p* < 0.001) and those aged 65 years and older (OR: 0.911, 95% CI: 0.864–0.961, *p* < 0.001). Furthermore, the results were consistent across gender groups, with females demonstrating an OR of 0.872 (95% CI: 0.821–0.925, *p* < 0.0001) and males an OR of 0.917 (95% CI: 0.860–0.978, *p* = 0.009). Consistency in the effect of DI-GM on frailty was also observed across races, PIR, smoking status, drinking status, recreational activity, and comorbidities including CVD, hyperlipidemia, COPD, and CKD (Figure 3).

### Mediation analyses

**Figure 4:**
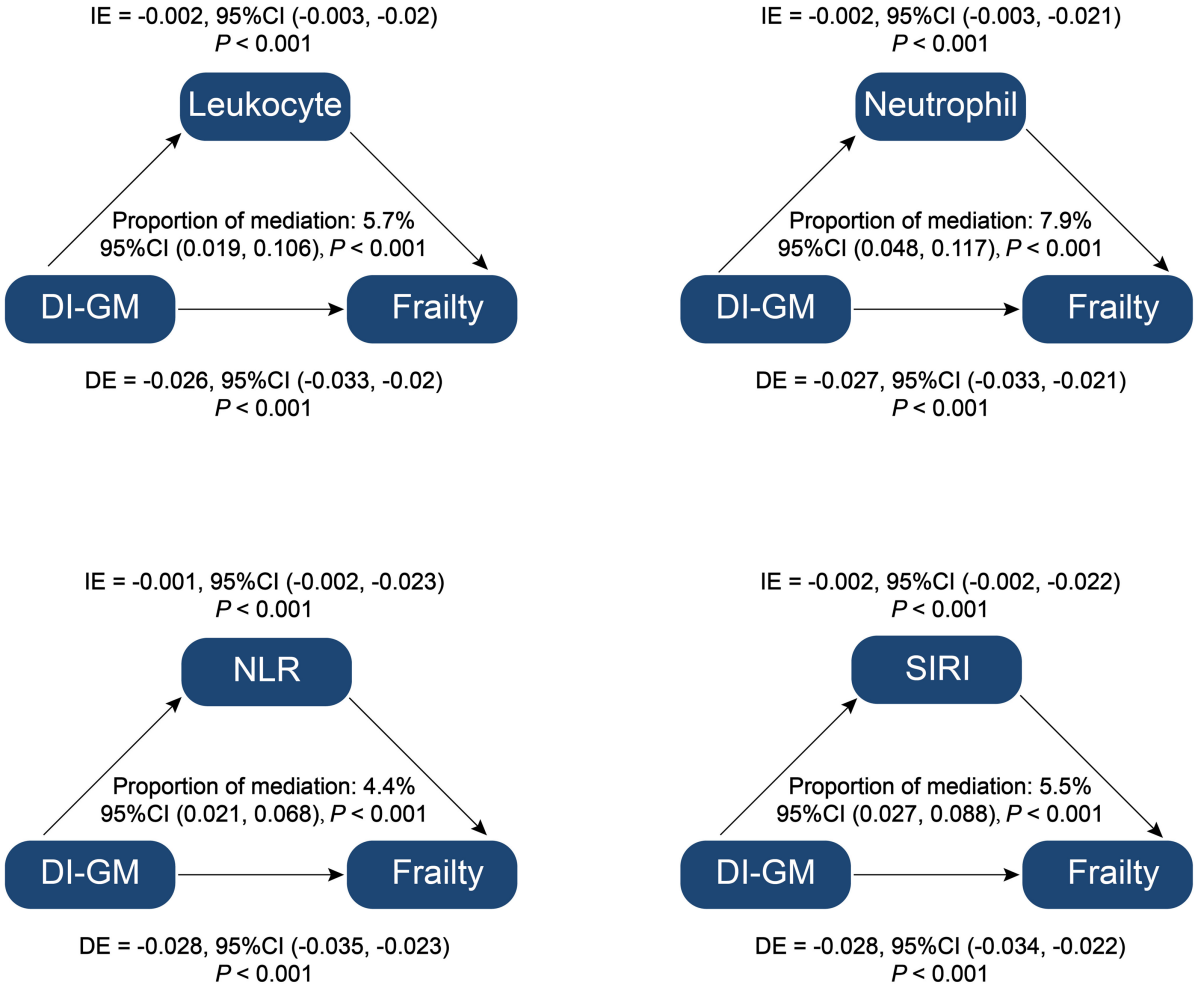
Mediation analysis of inflammation parameters on the association between Dietary Index for Gut Microbiota and Frailty. DI-GM, dietary index for gut microbiota; NLR, neutrophil to lymphocyte ratio; SIRI, systemic inflammation response index; IE, indirect effect; DE, direct effect; Proportion of mediation = IE/(DE + IE).

The mediation analysis assessed the roles of relevant inflammatory parameters from complete blood count, specifically leukocyte count, neutrophil count, NLR, and SIRI, in the relationship between DI-GM and frailty. The results indicated significant mediation effects for leukocyte count, neutrophil count, NLR and SIRI with mediation proportions of 5.7% (95% CI: 0.019–0.106, *p* < 0.001),7.9% (95% CI: 0.048–0.117, *p* < 0.001), 4.4% (95% CI: 0.021–0.068, *p* < 0.001), 5.5% (95% CI, 0.027–0.088, *p* < 0.001), respectively (Figure 4). These findings suggest that inflammatory markers play a vital mediating role in the association between DI-GM and frailty, highlighting the potential pathways through which dietary indices may impact frailty. Detailed information on the mediation effect of relevant inflammatory parameters from complete blood count is provided in Supplementary Table 3.

## Discussion

This study investigates DI-GM as a potential dietary exposure factor linked to frailty. Prior research has established that dietary patterns significantly influence gut microbiota composition, which is associated with various health outcomes, including frailty (8). Utilizing data from NHANES from 2007 to 2018, this cross-sectional analysis aims to elucidate the relationship between DI-GM and frailty risk, while exploring inflammatory markers as potential mediators of this association. A significant inverse relationship between DI-GM and frailty risk was shown, with part of this relationship mediated by inflammatory parameters. These results underscore the importance of dietary interventions as a promising avenue for frailty prevention strategies in this vulnerable population.

This study presents significant innovations in exploring the association between DI-GM and frailty risk among middle-aged and older adults, addressing a critical gap in the existing literature. While prior research has established the correlation between diet and gut microbiota, our robust population-based analysis utilizing NHANES data, spanning over 6 cycles, distinguishes our findings.

For the first time in a diverse human cohort, we demonstrate that higher DI-GM scores are significantly associated with reduced frailty risk, aligning with previous animal studies and human studies that indicated the potential of dietary interventions to modulate gut health and improve frailty outcomes (29, 30). Unlike earlier studies that primarily focused on individual dietary components or rehabilitation approaches, our research underscores the holistic perspective offered by dietary indices like the DI-GM (16, 31, 32). This advancement not only provides evidence of the long-term implications of dietary choices on frailty risk but also highlights the potential for targeted dietary interventions aimed at enhancing gut health as effective strategies for mitigating frailty in vulnerable populations.

In our study, mediation analysis revealed that inflammatory parameters significantly influenced the association between DI-GM and frailty, accounting for 4.4 to 7.9% of the total effect. This finding underscores the role of systemic inflammation as a mediating factor that may explain how DI-GM impacts frailty risk.

The DI-GM is a tool for assessing the impact of diet on gut microbiota, which may reflect the impact of dietary patterns on overall health (14). Studies have shown a strong association between dietary patterns and markers of inflammation. For example, diets with high inflammatory potential (such as high DII score) are often associated with higher white blood cell (WBC) count, neutrophil count and NLR (33). Elevated levels of these inflammatory markers reflect the role of diet in promoting systemic inflammation, which in turn may influence the onset and progression of frailty (34).

Systemic inflammation is an important pathophysiological mechanism of frailty. Studies have shown that inflammatory markers (such as white blood cell count, NLR and SIRI, etc.) are significantly increased in frail patients (20, 35–37). DI-GM regulates inflammation levels by influencing the composition and function of the gut microbiota. For example, a diet rich in fiber and polyphenols (with a high DI-GM score) promotes the proliferation of beneficial bacteria and inhibits the growth of harmful bacteria, thereby reducing the production of inflammatory mediators (14). This anti-inflammatory effect may reduce the risk of frailty by lowering levels of inflammatory markers (24). In addition, dysregulation of the gut microbiota, such as reduced microbiota diversity, is strongly associated with elevated inflammatory markers (35). Dietary patterns with low DI-GM scores, such as diets high in processed foods and red meat, may lead to dysregulation of the gut microbiota, which in turn promotes an inflammatory response (38). This inflammatory response not only affects gut health, but also can affect systemic health through the gut-brain axis and other pathways, accelerating the process of frailty (35).

This study has several limitations that require careful interpretation of the findings. Firstly, the reliance on cross-sectional data from NHANES constrains our ability to draw causal inferences regarding the relationship between DI-GM and frailty risk. This study was an observational design, and although we minimized the effect of confounders through multivariate adjustment and sensitivity analysis, potential reverse causation could not be completely excluded. Additionally, potential biases stemming from self-reported dietary intake may compromise the accuracy of our results, as participants may not accurately recall or report their dietary behaviors. While we attempted to control for various confounding factors, the presence of residual confounding cannot be entirely ruled out, which may influence the observed associations. Moreover, the lack of wet lab experiments limits our insights into the biological mechanisms that could explain the relationship between dietary factors and frailty. Finally, despite the substantial sample size enhancing statistical power, the generalizability of the findings may be restricted by demographic variations, underscoring the need for further validation in more diverse populations. Future research should prioritize longitudinal studies and experimental interventions to confirm these findings and investigate the causal mechanisms linking diet, gut microbiota, and frailty.

## Conclusion

This study shows a significant inverse association between DI-GM and frailty risk in middle-aged and older adults, with inflammatory parameters mediating part of this association. Our findings suggest that dietary interventions that increase DI-GM may reduce frailty risk by modulating inflammatory pathways. This suggests that public health strategies should promote high-fiber, fermented foods that support gut health, especially in middle-aged and older adults. In addition, these findings may guide future research on specific dietary patterns and their impact on inflammation to help health professionals develop effective frailty prevention strategies.

## Data Availability

The datasets presented in this study can be found in online repositories. The names of the repository/repositories and accession number(s) can be found at: The datasets supporting the conclusions of this article are available in the NHANES repository, https://www.cdc.gov/nchs/nhanes/index.htm.
