# Supplementary material for: Inflammatory parameters mediates the relationship between dietary index for gut microbiota and frailty in middle-aged and older adults in the United States: findings from a large-scale population-based study
Source: Front Nutr. 2025 Apr 16;12:1553467. doi: 10.3389/fnut.2025.1553467 (PMC12040679; doi:10.3389/fnut.2025.1553467)
Supplement: Supplementary file 1 [file Table_1.docx]

**Supplementary Table 1.** Components and scoring criteria of DI-GM in NHANES.

| Components of DI-GM | Included Foods within the Component | Scoring criteria |
| --- | --- | --- |
| Beneficial to gut microbiota | Avocados | For each component beneficial to gut microbiota, a score of 1 if consumption at or above the sex-specific median, else 0  The total score for beneficial components ranged from 0 to 10 |
|  | Broccoli |  |
|  | Chickpeas |  |
|  | Coffee |  |
|  | Cranberries |  |
|  | Fermented dairy (including yogurt, cheese, kefir, sour cream, buttermilk) |  |
|  | Fiber |  |
|  | Green tea |  |
|  | Soybean (including Soy milk, Tofu) |  |
|  | Whole grains (grains defined as whole grains, containing the entire grain kernel—the bran, germ, and endosperm) |  |
| Unfavorable to gut microbiota | High-fat diet (% energy) | 0 if consumption at or above 40% energy from fat, else 1  For each remaining component unfavorable to gut microbiota, a score of 0 if consumption at or above the sex-specific median, else 1  The total score for unfavorable components ranged from 0 to 4 |
|  | Processed meat (including frankfurters, sausages, corned beef, and luncheon meat that are made from beef, pork, or poultry) |  |
|  | Red meat (including beef, veal, pork, lamb, and game meat; excludes organ meat and cured meat) |  |
|  | Refined grains (refined grains that do not contain all of the components of the entire grain kernel) |  |

Abbreviations: DI-GM, dietary index for gut microbiota; NHANES, National Health and Nutrition Examination Survey.

**Supplementary Table 2.** Variables in the 49-Item Frailty Index and Their Respective Scorings Criteria in NHANES.

| **Item** | | **variable** | | **Scoring** | | |
| --- | --- | --- | --- | --- | --- | --- |
| **Cognition** | | - | | - | | |
| 1. experience confusion/memory problems | | pfq056, pfq057 | | yes=1; no=0 | | |
| **Dependence** | | - | | - | | |
| 2. managing money difficulty | | pfq060a, pfq061a | | no difficulty=0; | | |
|  |  |  |  | some difficulty=0.33; | | |
|  |  |  |  | much difficulty=0.66; | | |
|  |  |  |  | unable to do=1 | | |
| 3. walking for a quarter mile difficulty | | pfq060b, pfq061b | | the same to above | | |
| 4. walking up ten steps difficulty | | pfq060c, pfq061c | | the same to above | | |
| 5. stooping, crouching, kneeling difficulty | | pfq060d, pfq061d | | the same to above | | |
| 6. lifting or carrying difficulty | | pfq060e, pfq061e | | the same to above | | |
| 7. house chore difficulty | | pfq060f, pfq061f | | the same to above | | |
| 8. preparing meals difficulty | | pfq060g, pfq061g | | the same to above | | |
| 9. standingup from armless chair difficulty | | pfq060i, pfq061i | | the same to above | | |
| 10. getting in and out of bed difficulty | | pfq060j,pfq061j | | the same to above | | |
| 11. using fork, knife, drinking from cup difficulty | | pfq060k, pfq061k | | the same to above | | |
| 12. dressing yourself difficulty | | pfq060l, pfq061l | | the same to above | | |
| 13. standing for long periods difficulty | | pfq060m, pfq061m | | the same to above | | |
| 14. grasp/holding small objects difficulty | | pfq060p, pfq061p | | the same to above | | |
| 15. attending social event difficulty | | pfq060r, pfq061r | | the same to above | | |
| 16. leisure activity at home difficulty | | pfq060s, pfq061s | | the same to above | | |
| 17. push or pull large objects difficulty | pfq061t | | | | the same to above | |
| **Depressive Symptoms** | - | | | | - | |
| 18. have little interest in doing things | dpq010 | | | | **2007~** | |
|  |  |  |  |  | nearly every day = 1 | |
|  |  |  |  |  | more than half the days = 0.66 | |
|  |  |  |  |  | several days = 0.33 | |
|  |  |  |  |  |  | |
| 19. feeling down, depressed, or hopeless | dpq020, ciqd001, ciqd002 | | | | the same to above | |
| 20. trouble sleeping or sleeping too much | dpq030 | | | | **2007~** | |
|  |  |  |  |  | nearly every day = 1 | |
|  |  |  |  |  | more than half the days = 0.66 | |
|  |  |  |  |  | several days = 0.33 | |
| 21. feeling tired or having little energy | dpq040 | | | | nearly every day = 1 | |
|  |  |  |  |  | more than half the days = 0.66 | |
|  |  |  |  |  | several days = 0.33 | |
| 22. poor appetite or overeating | dpq050 | | | | **2007~** | |
|  |  |  |  |  | yes = 1 | |
|  |  |  |  |  | no = 0 | |
| 23. feeling bad about yourself | dpq060, ciqd029 | | | | the same to above | |
| 24. trouble concentrating on things | dpq070, ciqd043 | | | | the same to above | |
| **Comorbidities** | - | | | | - | |
| 25. doctor ever said you had arthritis | mcq160a | | | | yes = 1; no = 0 | |
| 26. ever told you had thyroid problem | mcq160i, mcd160m, mcq160m | | | | the same to above | |
| 27. ever told you had chronic bronchitis | mcq160k, mcq160p | | | | the same to above | |
| 28. ever told you had cancer or malignancy | mcq220 | | | | the same to above | |
| 29. ever told had congestive heart failure | mcq160b | | | | the same to above | |
| 30. ever told you had coronary heart disease | mcq160c | | | | the same to above | |
| 31. ever told you had angina/angina pectoris | mcq160d | | | | the same to above | |
| 32. ever told you had heart attack | mcq160e | | | | the same to above | |
| 33. ever told you had a stroke | mcq160f | | | | the same to above | |
| 34. ever told you had high blood pressure | bpq020 | | | | the same to above | |
| 35. doctor told you have diabetes | diq010 | | | | yes = 1; no =0; borderline=0.5 | |
| 36. ever told you had weak/failing kidneys | kiq020, kiq022 | | | | yes = 1; no =0 | |
| 37. urine leakage bother you? | kiq050 | | | | **2007~** | |
|  |  |  |  |  | greatly = 1 | |
|  |  |  |  |  | very much = 0.75 | |
|  |  |  |  |  | somewhat = 0.5 | |
|  |  |  |  |  | only a little = 0.25 | |
| **Hospital Utilization and Access to Care** | | | - | | | - |
| 38. general health condition | | | huq010 | | | excellent, very good, good = 0 |
|  |  |  |  |  |  | fair, poor = 1 |
| 39. health now compared with 1 year ago | | | huq020 | | | about the same, better = 0 |
|  |  |  |  |  |  | worse = 1 |
| 40. overnight hospital patient in last year | | | huq070, hud070, huq071 | | | yes = 1, no = 0 |
| 41. times receive healthcare over past year | | | huq050, huq051 | | | none = 0; 1-4 = 0.5; >=5 =1 |
| 42. number of prescription medicines taken | | | rxd030, rxduse, rxd295, rxdcount | | | no = 0; 1-4 = 0.5; >=5 =1 |
| **Physical Performance and Anthropometry** | | | - | | | - |
| 43. body mass index (kg/m^2) | | | bmxbmi | | | <18.5, ≥30 = 1 |
|  |  |  |  |  |  | 25–<30 = 0.5 |
|  |  |  |  |  |  | 18.5–25 = 0 |
| **Laboratory Values** | | | - | | | - |
| 44. glycohemoglobin(%) | | | lbxgh | | | 0%–5.7% = 0, >5.7% = 1 |
| 45. red blood cell count (million cells/ul) | | | lbxrbcsi | | | M: 4.7–6.1 = 0, Other = 1 |
|  |  |  |  |  |  | F: 4.2–5.4 = 0, Other = 1 |
| 46. hemoglobin (g/dl) | | | lbxhgb | | | M: 13.5–18 = 0, Other = 1 |
|  |  |  |  |  |  | F: 12–16 = 0, Other = 1 |
| 47. red cell distribution width (%) | | | lbxrdw | | | 11.6–14.6 = 0, Other = 1 |
| 48. lymphocyte percent (%) | | | lbxlypct | | | 20–40 = 0, Other = 1 |
| 49. segmented neutrophils percent (%) | | | lbxnepct | | | 40–80 = 0, Other = 1 |

**Supplementary Table 3.** Mediation Effects of relevant inflammatory parameters from complete blood count in the association between DI-GM and Frailty.

| **Mediator** | **Indirect effect** | **Direct effect** | **Total effect** | **Mediation proportions, % (95%CI)** |
| --- | --- | --- | --- | --- |
| **Leukocyte** | -0.002 (-0.003, -0.02) ^***^ | -0.026 (-0.033, -0.02) ^***^ | -0.028 (-0.034, -0.022) ^***^ | 5.7% (0.019, 0.106) ^***^ |
| **Neutrophil** | -0.002 (-0.003, -0.021) ^***^ | -0.027 (-0.033, -0.021) ^***^ | -0.03 (-0.035, -0.023) ^***^ | 7.9% (0.048, 0.117) ^***^ |
| **Monocyte** | -0.001 (-0.002, -0.023) ^***^ | -0.029 (-0.035, -0.023) ^***^ | -0.03 (-0.036, -0.024) ^***^ | 3% (0.012, 0.054) ^***^ |
| **Lymphocyte** | 0 (0, -0.023) | -0.03 (-0.035, -0.023) ^***^ | -0.03 (-0.035, -0.023) ^***^ | / |
| **Platelet** | 0 (0, -0.023) | -0.03 (-0.036, -0.023) ^***^ | -0.03 (-0.036, -0.023) ^***^ | / |
| **NLR** | -0.001 (-0.002, -0.023) ^***^ | -0.028 (-0.035, -0.023) ^***^ | -0.03 (-0.036, -0.024) ^***^ | 4.4% (0.021, 0.068) ^***^ |
| **MLR** | 0 (-0.001, -0.024) ^*^ | -0.029 (-0.035, -0.024) ^***^ | -0.03 (-0.036, -0.024) ^***^ | 1.6% (0.003, 0.031) ^*^ |
| **PLR** | 0 (0, -0.023) | -0.03 (-0.036, -0.023) ^***^ | -0.03 (-0.036, -0.023) ^***^ | / |
| **SII** | -0.001 (-0.002, -0.023) ^***^ | -0.029 (-0.035, -0.023) ^***^ | -0.03 (-0.036, -0.024) ^***^ | 2.9% (0.009, 0.055) ^***^ |
| **SIRI** | -0.002 (-0.002, -0.022) ^***^ | -0.028 (-0.034, -0.022) ^***^ | -0.03 (-0.036, -0.024) ^***^ | 5.5% (0.027, 0.088) ^***^ |

Abbreviations: MLR, monocyte to lymphocyte ratio; NLR, neutrophil to lymphocyte ratio; PLR, neutrophil to lymphocyte ratio; SII, systemic immune inflammation index; SIRI, system inflammation response index; ^*^ P < 0.05；^***^ P < 0.001
